# Trajectories of human brain functional connectome maturation across the birth transition

**DOI:** 10.1371/journal.pbio.3002909

**Published:** 2024-11-19

**Authors:** Lanxin Ji, Iris Menu, Amyn Majbri, Tanya Bhatia, Christopher J. Trentacosta, Moriah E. Thomason

**Affiliations:** 1 Department of Child and Adolescent Psychiatry, New York University School of Medicine, New York, New York State, United States of America; 2 Department of Psychology, Wayne State University, Detroit, Michigan, United States of America; 3 Department of Population Health, New York University School of Medicine, New York, New York State, United States of America; 4 Neuroscience Institute, New York University School of Medicine, New York, New York State, United States of America; Inserm U1208, FRANCE

## Abstract

Understanding the sequence and timing of brain functional network development at the beginning of human life is critically important from both normative and clinical perspectives. Yet, we presently lack rigorous examination of the longitudinal emergence of human brain functional networks over the birth transition. Leveraging a large, longitudinal perinatal functional magnetic resonance imaging (fMRI) data set, this study models developmental trajectories of brain functional networks spanning 25 to 55 weeks of post-conceptual gestational age (GA). The final sample includes 126 fetal scans (GA = 31.36 ± 3.83 weeks) and 58 infant scans (GA = 48.17 ± 3.73 weeks) from 140 unique subjects. In this study, we document the developmental changes of resting-state functional connectivity (RSFC) over the birth transition, evident at both network and graph levels. We observe that growth patterns are regionally specific, with some areas showing minimal RSFC changes, while others exhibit a dramatic increase at birth. Examples with birth-triggered dramatic change include RSFC within the subcortical network, within the superior frontal network, within the occipital-cerebellum joint network, as well as the cross-hemisphere RSFC between the bilateral sensorimotor networks and between the bilateral temporal network. Our graph analysis further emphasized the subcortical network as the only region of the brain exhibiting a significant increase in local efficiency around birth, while a concomitant gradual increase was found in global efficiency in sensorimotor and parietal-frontal regions throughout the fetal to neonatal period. This work unveils fundamental aspects of early brain development and lays the foundation for future work on the influence of environmental factors on this process.

## Abstract 2

How do the functional networks in the brain change at the beginning of human life? This longitudinal perinatal fMRI study of 140 babies reveals regionally specific changes in neural connectivity at the birth transition, with some areas showing minimal variation while others show increases in connectivity at birth.

## Introduction

The transition from the womb to the external environment requires rapid adaptation across multiple organ systems, including the brain [1]. Throughout gestation, rapid neural proliferation, migration, and even regression, occur alongside axonal growth and synaptogenesis [2,3]. After birth, the brain enters phase of dramatic outgrowth and expansion, with a surge in axonal myelination, dendritic arborization, and the rapid accrual of functional synaptic contacts [4]. Unveiling global connectional processes, during this early phase, is essential for understanding the emergence of brain functions and the origin of developmental disorders. In recent years, researchers have begun using resting-state functional magnetic resonance imaging (fMRI) to probe neural connections. FMRI detects fluctuations in blood oxygen level-dependent (BOLD) which are surrogates for neural activity. Correlations between BOLD signals arising from 2 distinct brain regions, known as resting-state functional connectivity (RSFC), indicate that the neurons of these regions are functionally connected. Groups of these functional connected regions then compromise brain networks. Studies using RSFC analyses have identified emerging resting-state networks in fetuses as early as the second trimester and in infants [1,5–8]. However, the massive reorganization of large-scale functional networks over the birth transition has yet to be studied longitudinally across birth.

Pioneering cross-sectional studies have utilized fetal brain fMRI to investigate RSFC prior to birth. First, RSFC in fetal brains was observed in bilateral occipital and bilateral frontal networks [9]. As gestational age advances towards term, intra-hemispheric, cross-hemispheric, and long-range RSFC strengthen [10–12]. Graph theoretical studies have shown that adult-like network topology also begins to be established during gestation [13,14]. In the adult human brain, the brain network exhibits small-world topology, characterized by a high clustering level and short path lengths for efficient, low-energy communication [15,16]. Highly connected regions within the network are known as “hubs.” In fetal functional networks, presence of small-world structure and hubs in sensorimotor regions has been reported [14,17,18]. As gestational age advances, there is a trend toward increased inter-module connections, suggesting greater network integration [18]. At the same time, modularity—reflecting how well a network can be divided into distinct, nonoverlapping parts—decreases, indicating reduced network segregation [17,18]. In brain graph analysis, segregation refers to the specialized functioning of distinct brain regions, whereas integration describes the coordinated activity across different brain networks. Moreover, fetal RSFC has been found to be sensitive to prenatal adverse exposure [19–21] and related to postnatal brain and behavior development [22–25]. The risk of neuropsychiatric disorders such as major depression, autism, and schizophrenia is also associated with neural development in the womb [26,27].

After birth, functional networks and network topology becomes more adult-like than the fetal period [28]. Primary networks are detected during neonatal period, including the primary visual cortex, bilateral sensorimotor area, bilateral auditory cortex, a network encompassing the precuneus area, lateral parietal cortex, and the cerebellum. In contrast, higher-order networks, such as the default mode network and the executive control network, appear incomplete and divided, suggesting that they are still in formation process. However, functional connectivity between isolated parcels of these networks consistently increases in the first 2 years of life [5,29]. Meanwhile, functional hubs spread into primary sensorimotor, visual regions, and Wernicke’s area [30]. Small-world architecture also continues to develop after birth, with a remarkable improvement in whole brain wiring, becoming more stable by approximately 2 years old [31]. With development, the balance between network segregation and integration tends to be optimized, leading to a better-organized connectome architecture [32].

Based on existing fetal and infant fMRI findings, it is hypothesized that the brain connectional development from the mid-gestation through early infancy follows the “local to distributed” developmental pattern [32–34]. This hypothesis suggests a shift from the rapid development of local primary clusters and short-range connections before birth, to the growth of long-range connections after birth, which enhances the efficiency in global and local information transfer ability [33]. While previous studies have provided rich analyses of the fetal and, separately, infant period, the absence of a rigorous examination of the brain functional networks across the birth transition represents a major gap in the field. A recent study, the first to use both fetal and infant scans, shows a significant increase in both intra- and inter-network RSFC from the 30th to the 46th week postmenstrual age. However, this prior study included repeated scans across the birth transition from a small sample (*n* = 29), and the analysis was restricted to 3 higher-order networks of default mode network, salience network, and the executive control network [35].

In our study, we leverage an existing perinatal longitudinal fMRI data set spanning 25 to 55 post-conceptual gestational weeks, aiming to provide a comprehensive evaluation of RSFC development across the whole brain throughout the birth transition. Our goal is to understand not only whether brain networks change with age but also the shape and form of the developmental trajectory. In addition, we examine the RSFC changes within the fetal and infant periods separately, aiming to confirming findings from previous studies. We hypothesize that primary functional networks, such as the parietal, occipital, and subcortical regions, will show a sharp increase in RSFC both within these networks and with others. This increase, along with greater network efficiency, is expected as the brain prepares for external stimuli before birth and adapts to them afterward. This study is fundamental to understanding the brain-based origins of human behavior and, also, critical in establishing normative models that can provide a crucial reference for future studies of brain network connectional architecture, and timing of development, in clinical research samples.

## Results

### Significant development of functional systems on a global scale

**Fig 1:**
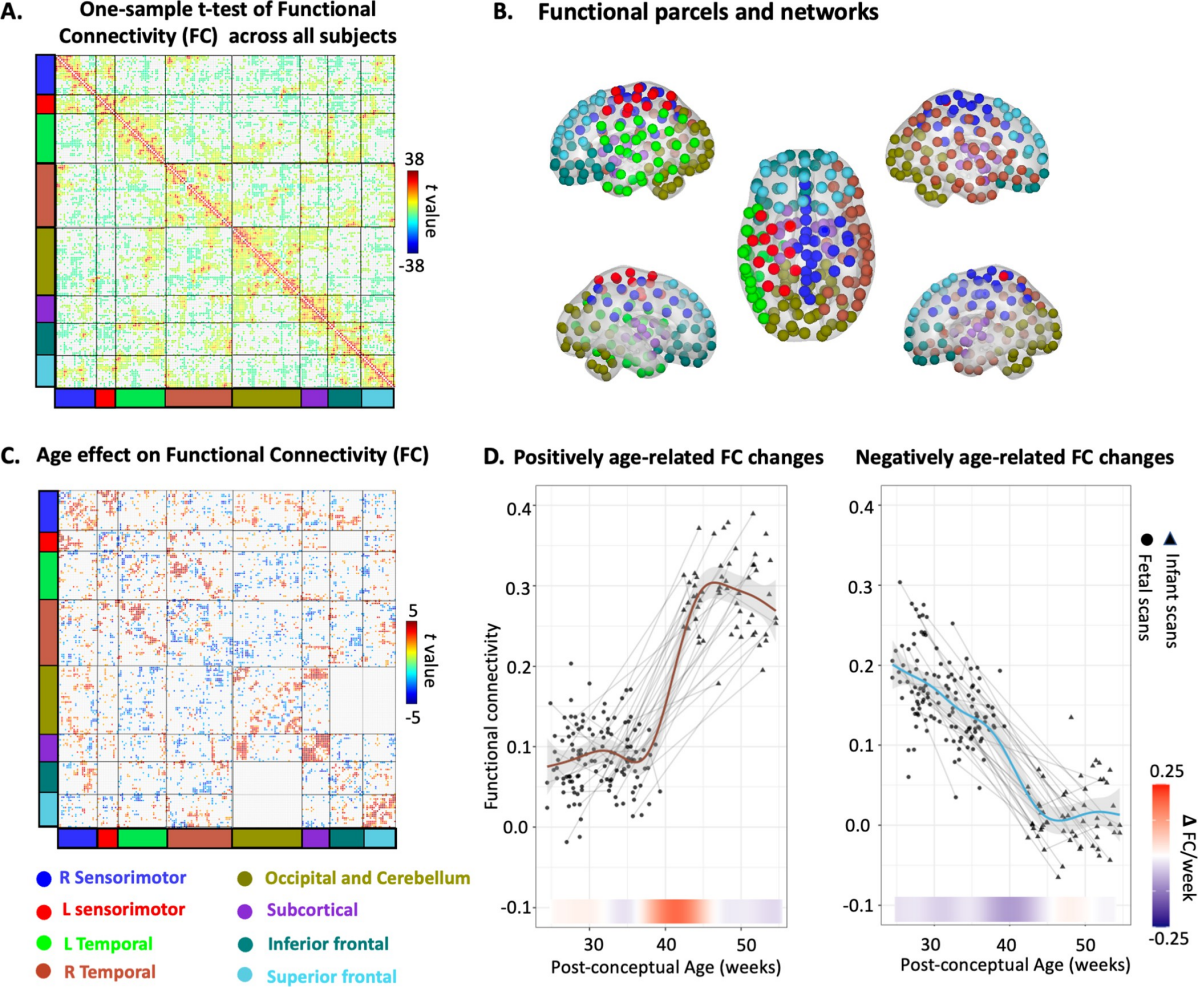
Longitudinal trajectories of functional network development across the birth transition. (A) One-sample *t* test on RSFC across all subjects. Stronger RSFC within networks affirms validity of the network clustering algorithm. (B) Functional parcels and networks. (C) Age effect on the ROI-to-ROI functional connectivity. (D) Maturation trajectory and rates of change of the average age-related RSFC. The growth trajectory of “positive age-related RSFC” (left) represents the average RSFC across all connectivity edges that show a significant positive age effect, as colored in red in Fig 1C. Similarly, the “negative age-related RSFC” represents the average RSFC for all connectivity edges with a significant negative age effect, shown in blue in the matrix in Fig 1C. The shaded gray area represents 95% confidence intervals. Lines indicate longitudinal data from the same participant scanned at multiple time points. Data points from fetuses or infants are indicated by either circle or triangle. The change rate of RSFC is presented under the plot (navy blue to red). The data used to generate this figure can be found in S1 Data. ROI, region of interest; RSFC, resting-state functional connectivity.

This study analyzed a total of 203 fMRI scans, covering a gestational age (GA) range of 25 to 55 weeks post-conception. The data set comprised 140 fetal and 63 infant scans, all acquired by the senior authors on a single Siemens Verio 3T system between 2013 and 2018 as part of the Perinatal Imaging of Neural Connectivity (PINC) project at Wayne State University (WSU). After quality control, the final sample for the functional connectivity analysis includes 126 fetal scans (GA = 31.36 ± 3.83 weeks) and 58 infant scans (GA = 48.17 ± 3.73 weeks) from 140 unique subjects. Preprocessed fMRI data from both fetal and infant scans were used to create a data-driven, group-balanced functional atlas, consisting of 195 functional parcels and 8 networks within the gray matter (**Fig 1B**). The functional atlas and the gray matter mask are provided in Supporting information (SI) S1 Fig. For each region of interest (ROI), the average time series across voxels was calculated, and these time series were used to construct a 195 × 195 RSFC matrix for each scan session. A one-sample *t* test was conducted across all available scans to show the group mean ROI-by-ROI RSFC matrix (**Fig 1A**). Age-related RSFC were then isolated by regression models controlling for repeated measures. Longitudinal Combat [36] were further applied to harmonize the fetal and infant RSFC data scanned using different sequences.

Subsequently, we examined the developmental trajectory of brain RSFC at the global average level by averaging the output RSFC exhibiting significant positive or negative associations with age to derive growth plots. The growth trajectory was modeled with the generalized addictive mixed-effect models (GAMMs), following the equation RSFC ~ 1 + s(Age) + (1|ID). To accurately quantify periods of significant developmental differences and estimate the developmental rate, we analyzed the local slope (first derivative) of age-related changes across all ages at 1/5th-week intervals for all nonlinear GAMM models.

These age-related RSFC changes involve widely distributed networks (**Fig 1C**). Positive age-related changes followed a nonlinear trajectory, with a notable sharp increase occurring at the birth transition (**Fig 1D**). A reverse U-shaped pattern in RSFC was observed within the fetal stage, peaking at approximately 30 weeks. On the other hand, the negative age-related RSFC showed constant decrease prior to birth and became relatively stable in early infancy.

### Differential timing of maturation across individual functional brain networks

**Fig 2:**
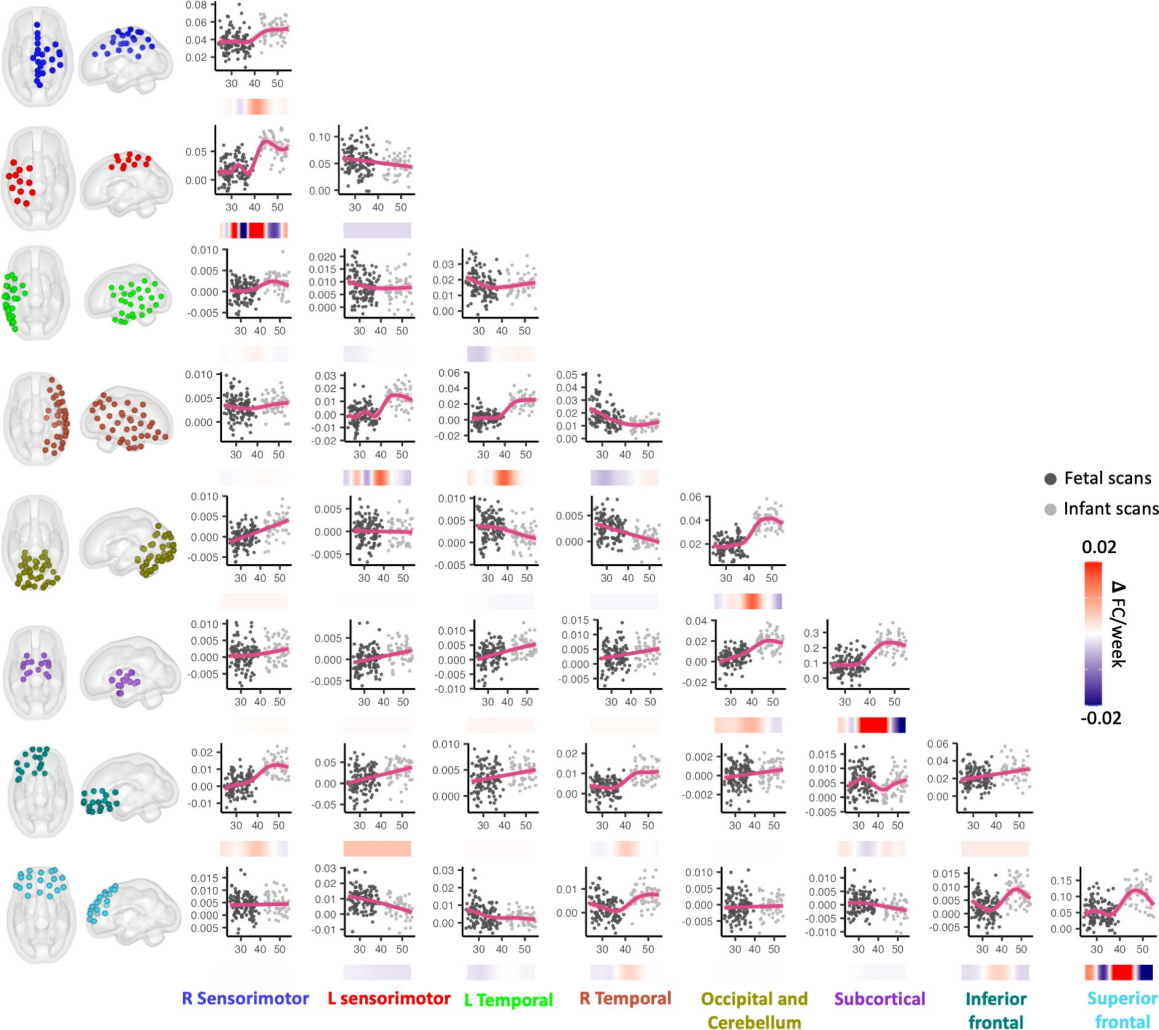
Maturation trajectory and rates of change of the pairwise within- and between-network functional connectivity. The shaded pink area represents 95% confidence intervals. Data points from fetuses or infants are indicated by either light gray or dark gray. The change rate of RSFC is presented under each plot (navy blue to red). The data used to generate the network-level results can be found in S2 Data. RSFC, resting-state functional connectivity.

To understand the development patterns for each network, we next conducted GAMM analyses on each within- and between-network connectivity across 8 global networks (*N* = 36 possible associations), using the same approach as described above. The network-level RSFC was calculated by averaging the ROI-by-ROI RSFC values that showed significant positive or negative associations with age. We did not separate the positive and negative effects in the plots, as we believe the dominant effect at the network level is more meaningful. This analysis revealed that there is a trend for increase in majority of RSFC; however, the shape of the growth curve varies across network (**Fig 2**). A growth pattern of a nonlinear sigmoid shape was highlighted in connections within the subcortical network, within the superior frontal network, within the occipital-cerebellum joint network, as well as the cross-hemisphere RSFC between the bilateral sensorimotor networks and between the bilateral temporal network (**Fig 2**). Detailed statistical results of the GAMMs are provided at the S1 Table.

### Graph features develop throughout the birth transition period

Based on RSFC matrices, we further estimated the graph theory measures of global efficiency (GE) and local efficiency (LE) for each subject. For each ROI, we defined global efficiency as the average inverse shortest path distance from node n to all other nodes in the graph, and local efficiency as the average global efficiency of the neighboring subgraph of node n. We selected global and local efficiency because: (1) they are fundamental metrics of network properties—global efficiency reflects the effectiveness of information exchange across the entire network, indicating its overall connectedness, while local efficiency measures the efficiency of information transfer among neighboring regions, providing insight into local network integration; and (2) both metrics can be evaluated at the ROI level. We employed the same GAMM models used in the RSFC analysis to examine the growth trajectories of global efficiency and local efficiency.

**Fig 3:**
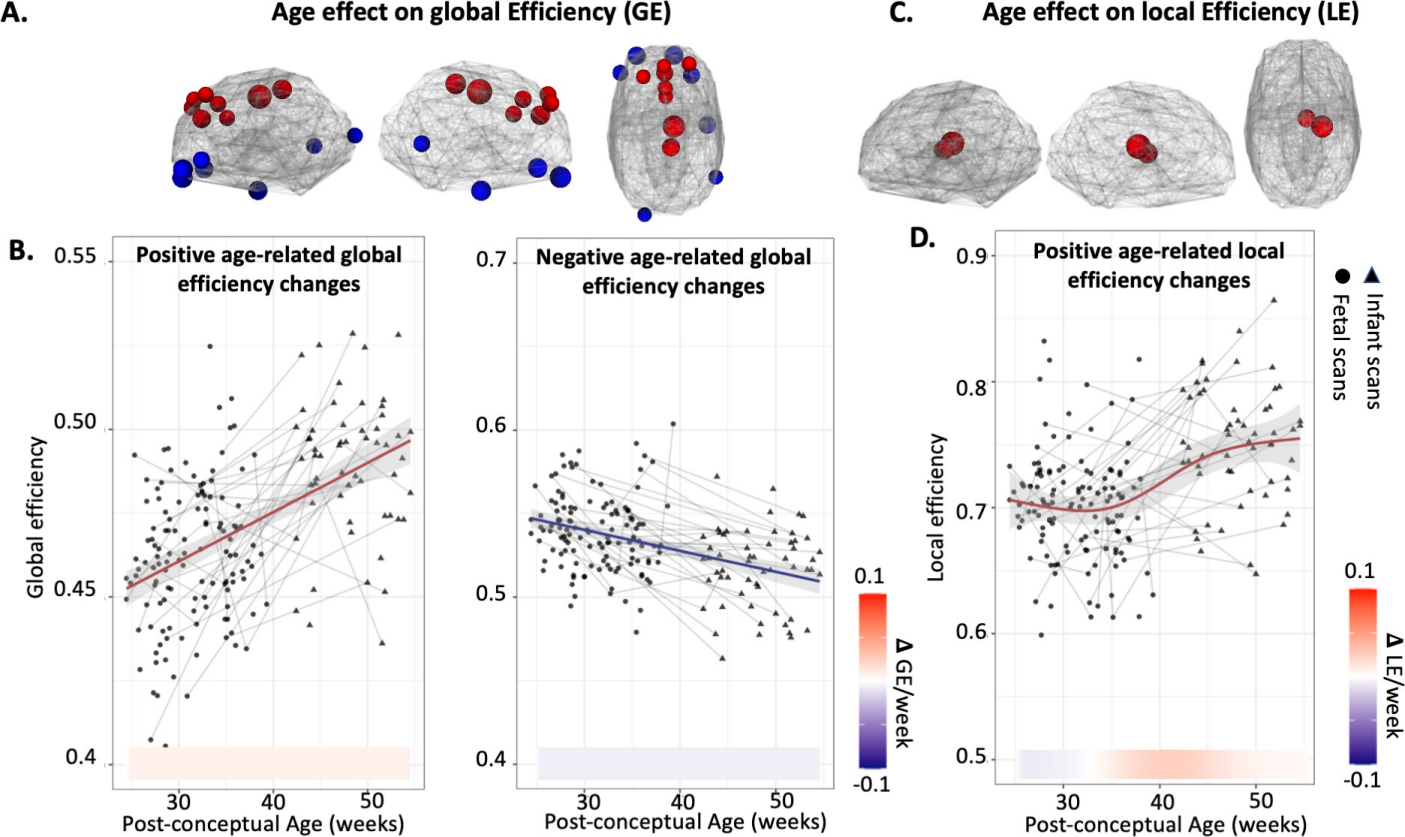
Changes in graph parameters over the birth transition. (A) Nodes showing significant age-related changes in GE, where red indicates increased GE with age, while blue indicates decreased GE with age. (B) Developmental trajectories of GE and the rates of change. (C) Nodes showing significant age-related increase in LE. (D) Developmental trajectories of LE and the rates of change. For the trajectory plots in (B) and (D), shaded gray area represents 95% confidence intervals. Lines indicate longitudinal data from the same participant scanned at multiple time points. Data points from fetuses or infants are indicated by either circle or triangle. The change rate of RSFC is presented under the plot. The data used to generate the graph measure results can be found in S1 Data. GE, global efficiency; LE, local efficiency; RSFC, resting-state functional connectivity.

Significant developmental effects were seen in multiple brain connectome graph features. Specifically, the global efficiency in supplementary motor and anterior partial regions increased with age, while the global efficiency in bilateral inferior frontal and a node in right temporal region decreased with age (**Fig 3A**). It is noteworthy that we did not observe an inflection point in the growth curve of global efficiency (**Fig 3B**). Local efficiency of the thalamus increased (**Fig 3C**) as age advanced, with a sigmoid shape of growth curve (**Fig 3D**). The peak increase of the subcortical local efficiency between 35th and 45th GA weeks echoes back to the within-network RSFC change as shown in **Fig 2**. Interestingly, we did not find significantly decreased local efficiency in any region.

### Overlapping and discrete changes across the entire birth transition period versus fetal or infant periods only

In follow-up analyses, we repeated age-regression procedures described above, within fetal and infant data sets, separately. These secondary analyses served to isolate significant RSFC developmental patterns that may be present within each period, but masked when evaluating longer durations, here, also, spanning birth.

**Fig 4:**
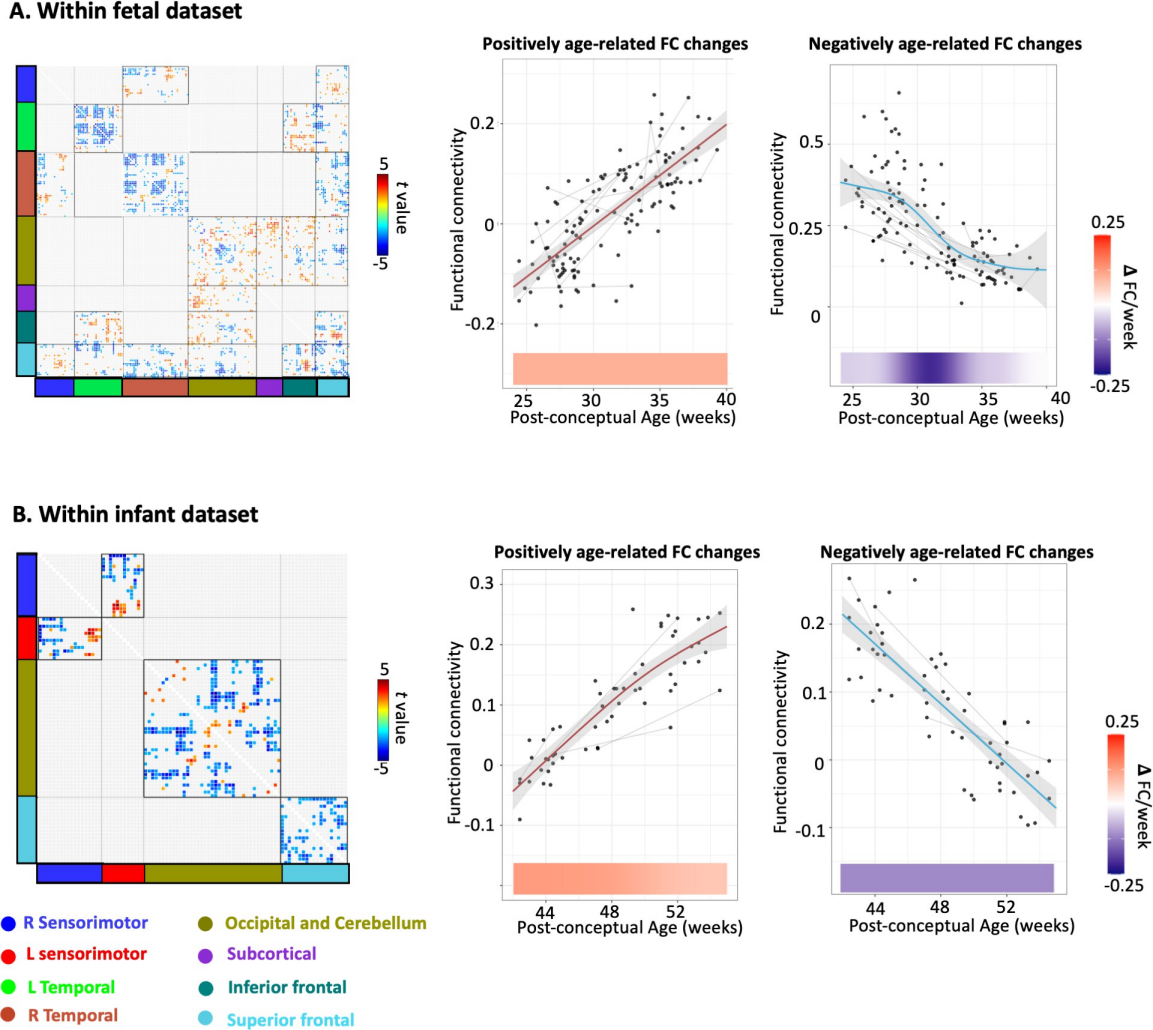
Age-related change in functional connectivity evaluated separately within fetal (A) and infant data sets (B). Age effect on the ROI-to-ROI functional connectivity and the maturation trajectory of the average age-related RSFC are presented side by side. The shaded gray area represents 95% confidence intervals. Lines indicate longitudinal data from the same participant scanned at multiple time points. The change rate of RSFC is presented under the plot (navy blue to red). The data used to generate this figure can be found in S1 Data. ROI, region of interest; RSFC, resting-state functional connectivity.

Interestingly, both overlapping and discrete changes are found between the analysis of the entire birth transition period and the examination of separate fetal or infant periods (**Fig 4**). Prior to birth, increased RSFC primarily involved the occipital-cerebellar network, including both its internal connections and its interactions with the subcortical and inferior frontal networks (**Fig 4A**). RSFC of these networks exhibited an almost linear increase through the fetal period. On the hand, decreased RSFC with age was observed in RSFC related to the superior fontal network and both left and right temporal network (**Fig 4A**). In our infant data set, age effect was primarily observed in 4 networks: the right and left sensory motor network, the occipital-cerebellar network, and the superior frontal network. Both increased and decreased RSFC were seen between the left and right sensorimotor network and within the occipital-cerebellar network, while only decreased RSFC was seen within the superior frontal network (**Fig 4B**).

## Discussion

Understanding the timing and sequence of brain network development over the birth transition is crucial. Previous studies have begun to isolate perinatal functional brain networks, but these studies are mostly confined to the fetal period or the infant period separately. Thus, we lacked knowledge about the maturation pattern of functional connectivity across birth. Utilizing a relatively large longitudinal perinatal data set and nonlinear modeling, this study represents, to our knowledge, the first to map the growth trajectory of global functional neural network across birth. Our results demonstrate a rapid surge in functional connectivity at birth on a global scale, probably reflecting neural processes that support the brain’s transition to the external world. Neural changes that accompany the birth transition are not uniform across the brain. Instead, changes in RSFC are exhibited by specific networks, with specific trajectories and magnitude of change. As examples, strengthened RSFC around birth is seen within the subcortical network, the superior frontal network, the occipital-cerebellum joint network, and both cross-hemispheric RSFC between bilateral sensorimotor networks and temporal networks.

Significant RSFC changes in subcortical regions around birth were one notable observed effect. Age-related changes were observed in the subcortical network in both RSFC and local efficiency analyses, the latter of which reflects the communication efficiency within the neighboring nodes. In fact, the subcortical network stood out as the only region of the brain that showed significant increase in local efficiency over this period. In the brain, the subcortical network represents a central hub, relaying nearly all incoming and outgoing information to and from the cortex and mediating cortico-cortical communication [37]. A recent fetal study shows that RSFC of the thalamus, a subcortical structure, develops prior to birth [38], and at the time of birth, this structure is already connected with the entire primary cortex [39]. Our results show that there is dramatic change in subcortical connectional architecture flanking the birth transition that temporally aligns with demands on the brain to process and integrate new kinds of information at this developmental transition.

Similarly, rapid increase in RSFC was observed across bilateral sensorimotor regions over the period leading up to and following human birth. Sensorimotor network represents one of the first systems of the brain to develop, and here, for the first time, we characterize the nature of change in this system during this transformational developmental stage. Our earlier work has established that the sensorimotor network emerges during the fetal period and that dynamic features of this network can predict infant motor behavior [22]. The sensorimotor network has also been isolated in preterm and term infants studied between 32 and 45 postmenstrual weeks [40]. Hubs and rich-club structure are also evident in primary motor and sensory regions by 31 weeks GA [30]. In this study, we show the dramatic development of the sensorimotor network across birth. Our results likely reflect the neural pruning and strengthening process, preparing the brain to respond to the abundant sensory and motor stimuli from the external environment prior to and upon birth. This aligns with our previous observation that infant motor development at 7 months is linked to prenatal RSFC development of the motor cortex [24].

It was notable that RSFC with the superior frontal cortex and its RSFC with the inferior frontal cortex showed rapid increase across birth as this was not entirely predicted. That is, function of the frontal lobe is traditionally believed to mature later; however, it is known that prefrontal neural differentiation of this region begins as early as 17 to 50 GA weeks in humans [41]. In fetal RSFC studies, connectivity in the frontal regions begins to emerge as early as 26 weeks [12]. Stable signal components are also detected in the frontal areas [9]. Notably, the RSFC within the superior frontal network showed a decreasing trend within either fetal or infant data in our secondary analysis within separated periods, suggesting that the increase across the birth is mainly driven by the birth event. Intriguingly, the rapid changes are observed only in the superior part of the frontal lobe but not in the inferior part. We infer that this may indicate potential variations within the frontal network.

It is noteworthy that select regions showed consistent decrease in RSFC with age (**Fig 1**). According to the “local to distributed” developmental hypothesis, the whole-brain network seems to lean toward segregation enforcement during the prenatal stage, which is supported by excess of short-range connectivity [33]. As age advances, decreased modularity and increased inter-module connection strength were detected, resulting in enhancement of the network integration process [33,42]. Actually, we did not see a significant difference in the actual ROI-to-ROI distance between the positively age-related RSFC and the negatively age-related RSFC (see the distribution of RSFC on Euclidean distance in S2 Fig). Thus, we infer that the decrease in RSFC might not only correspond to a developmental shift from short-range to long-range connections, but could also be related to the network reorganization, potentially contributing to higher efficiency. This reorganization could occur alongside the emergence of billions of new synaptic junctions and an overproduction of macroscopic connections around the time of birth. Indeed, similarly to the developmental rate shown in the negatively age-related RSFC, global efficiency is gradually strengthened throughout the birth transition, with a constant rate. This is intriguing, as the developmental trajectory of global efficiency does not follow the same pattern as that of positively age-related RSFC with a dramatic change around birth. We infer the reason is that (1) the optimization of the network efficiency lags behind the establishment of single connections, as it may involve multiple rounds of forward-backward feedback; (2) the global efficiency, which is the reciprocal of shortest path between 2 nodes, is not only related to the establishment or strengthening of connections but also to the elimination of redundant connections, which is reflected by our findings of decreased RSFC with age. Our results of regions with enhanced global efficiency replicated previous findings on emerging hubs in primary sensorimotor regions in neonates [30].

In our secondary analysis using only the fetal or the infant data set, both overlapping and discrete changes were found compared to the analysis of the entire birth transition period. For example, a consistent increase in RSFC was noted within the occipital-cerebellum network both during the fetal period and throughout the birth transition. In contrast, RSFC within the superior frontal network decreased during the fetal or infant period but displayed a dramatic increase across birth. These findings highlight the importance of leveraging longitudinal data spanning birth, as the developmental patterns may be specific to the short window surrounding birth but have largely been overlooked in studies utilizing single time points. Future studies that replicate and extend work with specific brain networks are warranted.

The limitations of our study warrant mention. Mapping the trajectory of brain connectome development across the birth transition presents unique challenges, particularly concerning potential variability introduced by different scanning conditions (in-utero versus ex-utero) and preprocessing strategies. There are numerous differences to fetal images in comparison to infant data due to a number of factors, including both fetal and maternal sources of noise. Frequent and large-scale fetal movements, as well as surrounding motion from maternal respiration, amniotic fluid flow, and arterial pulsation, can lead to temporally varying patterns of spatial distortion and signal changes. Moreover, adjacent maternal tissues and structures, such as the bowel, can also cause localized areas of distortion or signal loss. There are also localized differences due to spatial distances between the fetal brain and the static receive coil placed on the maternal abdomen. Overall, fetal data inherently suffers from significantly lower temporal signal-to-noise ratio (tSNR) compared to neonatal data (S3 Fig). Due to the factors mentioned above, fetal images are likely to exhibit spatial variability, which could impact RSFC estimations. However, unfortunately, there is also no ground truth to test the effect of imaging conditions as we do not have a constant variable to serve as a reference. On one hand, we convinced ourselves that the effect does not attribute to image quality, as we observed diverse growth trajectories across networks rather than a global uniform increase. On the other hand, we also confirmed that there is no correlation between RSFC and the motion parameters as provided in the S4 Fig. In addition, we implemented 2 strategies in our analysis to mitigate the impact of different tSNR between data sets: (1) In our RSFC analysis, we used the z-values of RSFC for all subsequent analyses to correct for the variance differences. (2) We applied an additional harmonization approach to eliminate the effects of this variance difference before conducting our statistical analyses. Another important limitation to note is that fMRI is an indirect measure of neural activity, as it relies on blood oxygen levels. Therefore, the RSFC findings observed in this study may be influenced by non-neurological factors involved in the hemodynamic response, such as cerebral blood flow and the cerebral metabolic rate of oxygen (CMRO2). At birth, there are dramatic changes in brain physiology, particularly in cerebral blood flow [43]. Consequently, the sharp increase in RSFC observed around birth could be a combined result of changes in both neural activity and brain physiology.

## Conclusion

The present study offers new knowledge on brain RSFC development across birth using a one-of-the-kind perinatal longitudinal data set. Findings suggest that RSFC develops at varied rate and exhibits diverse shape across the total brain network, including both increasing or decreasing trends, and gradual linear increases or rapid surges around birth. The subcortical network, sensorimotor network, and the superior frontal network stand out as undergoing rapid reorganization during this developmental stage. This work lays the foundation for future work regarding the maturational timing of brain functional networks spanning the perinatal period. Extending from this work, one can imagine further studies examining how factors such as sex, prematurity and prenatal adversity interact with the timing and growth patterns of children’s brain network development.

## Materials and methods

### Participants

This study used in total of 203 fMRI scans (25 to 55 weeks post-conceptual GA), consisting of 140 fetal and 63 infant scans, collected on a single Siemens Verio 3T system, between 2013 and 2018, as part of the Perinatal Imaging of Neural Connectivity (PINC) project at Wayne State University (WSU). Eligibility criteria included singleton pregnancy, maternal age 18 to 40, no suspected central nervous system abnormality as determined by 20-week ultrasound, and no contraindication for MRI. The WSU Institutional Review Board approved all study procedures, and informed written consent was provided by participating pregnant people. MRI visits occurred when the fetuses were between 20 and 40 weeks GA, and infants were between 0 and 3.5 months at follow-up. Select cases were scanned 1 to 3× longitudinally, with peaks centered at 27 weeks, 36 weeks, and 2 months.

### FMRI data acquisition

#### Fetal fMRI

Fetal fMRI was acquired using a 3T Siemens Verio 70 cm open-bore system with an abdominal 4-channel Siemens Flex coil. FMRI data were attained with either of the following scanning parameters: (1) 12-min single-echo fMRI: TR/TE = 2,000/30 ms; resolution = 3.4 × 3.4 × 4 mm^3,^ flip angle: 80 degrees. (2) 12-min multi-echo (ME) fMRI: TR = 2,000 ms; TE = 18, 34, 50 ms (3 echoes); flip angle: 83 degrees; voxel size: 3.5 × 3.5 × 3.5 mm^3^. Scans were repeated when time permitted.

#### Infant fMRI

Infants were scanned on the same 3T Siemens Verio 70 cm open-bore system with a 32-channel head coil, using one of 3 sets of acquisition parameters, as detailed below. (1) 12-min ME-fMRI: TR = 2,000 ms; TE = 13, 26, 39 ms; flip-angle: 83 degrees; slice-gap: none; voxel-size: 3.5 × 3.5 × 3.5 mm^3^; matrix-size: 64 × 64 × 39 voxels. (2) 12-min multi-band (MB) ME-fMRI scan: TR = 1,500 ms; TE = 15, 31, 46 ms; flip-angle: 83 degrees; slice-gap: none; voxel-size: 2.9 × 2.9 × 2.9 mm^3^; matrix-size: 64 × 64 × 48 voxels, multi-band factor = 2. (3) 7-min MB ME fMRI scan: TR = 1,000 ms; TE = 14.6, 36.68, 58.76 ms; flip-angle: 52; slice-gap: none; voxel-size: 2.5 × 2.5 × 2.5 mm^3^; matrix- size: 80 × 80 × 44 voxels, multi-band factor = 4.

### FMRI preprocessing

#### Fetal fMRI

Preprocessing began with automatic fetal brain segmentation using deep learning. A single mask was hand drawn onto a reference frame within a section of low motion for each acquired run. For every volume in the time series, a convolutional neural network (CNN)-trained model automatically segmented the brain from the maternal compartment, generating a rough 4D mask for the entire time series [44]. The brain was then extracted using this rough mask for motion estimation using FSL v5.0 mcflirt [45] where transformation matrices for mapping each volume to the reference frame were generated. We call the CNN-generated mask the “rough mask” because the CNN segmentation cannot perform perfectly on all volumes, with frequent occurrence of minor extra tissue or brain tissue loss (see example as provided by [44]). However, the rough mask is necessary and helpful here because motion estimation requires a clear background without maternal tissue. We then applied the inverted transformation matrices to the manual drawn mask to generate a precise, individualized 4D mask. The raw data were then masked again using this precise 4D mask. In a final step, we repeated these steps to derive a more refined transformation matrices that served as the basis for the final smartly generated semi-automated 4D mask for precise brain extraction from the raw time series data. Motion parameters were computed for the final masked series to be used in subsequent analyses. Following this, a three-step approach to censoring was applied. First, frames with a Sørensen–Dice coefficient (DC) below 0.9 between the frame and the reference frame (the one used for manual brain tracing) were censored. Next, framewise displacements (FDs) were calculated and any frame above 2 standard deviations above the mean (FD > 1.5 mm) across all inputted runs was censored. Finally, root-mean-square of voxel-wise differentiated signal (DVARS, [46]) were calculated and any volumes with DVARS greater than 2 standard deviations above the mean (DVARS > 132.69) across all inputted runs was censored. Further, participants with fewer than 105 low-motion frames were excluded.

Subsequent preprocessing steps included optimal combination across echoes using T2*-based weighting [47] (for multi-echo data), manual reorientation using SPM [48], normalization to the developing Human Connectome Project (dHCP) 34-week preterm infant template [49], individual-level Independent Component Analysis (ICA) denoising [50], smoothing with a 3 mm kernel, and CompCor denoising [51] implemented in the Connectivity RSFC Toolbox (CONN21.a) [52]. Specifically, for normalization, a mapping between functional native space and the template space was constructed by concatenating a linear transformation between the functional scan and the age-matched fetal template [53] estimated by SPM12, and a sequence of nonlinear transformations between templates of adjacent ages (e.g., 24 and 23, 25 and 24) estimated by Advanced Normalization Tools (ANTs v2.4.3) [54]. This gradual alignment will minimize the risk of gross misalignments due to differences in brain topology across GA.

#### Infant fMRI

Infant brain preprocessing followed prior published procedures [55], beginning with FSL’s Brain Extraction Tool (BET, [56]) and motion correction using “FSL mcflirt.” Volumes with root-mean-square of voxel-wise differentiated signal (DVARS) greater than 50 were marked as outliers (censored frames). One frame before and 2 frames after these volumes were also censored, as recommended in prior literature [57]. Motion-corrected data were subsequently combined across echoes, denoised using TE-Dependent Analysis (Tedana v22.0.1, [58,59]), normalized to the 34-week preterm infant template, denoised with the CompCor algorithm in CONN, and smoothed using a 3 mm kernel. Normalization used the stepwise approach described above for fetal scans.

### FMRI quality assurance

The preprocessed data was manually inspected by the first author, LJ. During preprocessing, subjects were excluded under the following circumstances: (1) frames fewer than 105 (*N* = 3 fetal scans and 1 infant scan); (2) incomplete brain coverage or severe distortion (*N* = 5 fetal scans and 2 infant scans); (3) normalization failure (*N* = 3 fetal scans and 2 infant scans); (4) high motion (mean translational displacement or rotation >0.5 or maximum >1 after censoring; *N* = 3 fetal scans). The final sample consisted of 126 fetal scans (GA = 31.36 ± 3.83 weeks) and 58 infant scans (GA = 48.17 ± 3.73 weeks) from 140 unique subjects. Of these, 108 subjects completed 1 scan, 21 completed 2 scans, 10 completed 3 scans, and 1 subject completed 4 scans (also see distribution of scans across age at S5 Fig). Detailed demographic characteristics of the final sample are provided in **Table 1**. We provide the scanning parameters and the quality control measure, DVARS, of the final sample post motion correction in **Table 2**. Additional quality control analyses showed that the correlation of RSFC-retained motion did not align with the anatomical distances among any ROI pair (see S4 Fig), suggesting that the relationship between motion, RSFC, and Euclidian distance is negligible [60].

**Table 1:** Participant characteristics.

	Subjects (*n* = 140)
Birth outcomes, *M (SD)*	
Fetal age at birth (weeks)	38.45 (2.43)
Birth weight (g) *	3,018.07 (644.33)
Sex, *n (%)*	
Female	79 (56%)
Male	61 (44%)
Maternal Ethnicity, *n (%)*	
African American/Black	114 (81%)
Asian American	1 (1%)
Caucasian/White	8 (6%)
Bi-racial	16 (11%)
Other	1 (1%)

* Birth weight of 128 subjects is available.

**Table 2:** Sequence descriptions and image quality control.

Sequence	No. of scans	TR (ms)	TE (ms)	Voxel dimensions (mm^3^)	Matrix size (voxel)	Flip angle (°)	Multi-band factor	Mean DVARS*	Max DVARS*
fetal (i)	75	2,000	30	3.4 × 3.4 × 4	96 × 96 × 25	80	NA	33.74 ± 9.10	95.59 ± 38.31
fetal (ii)	51	2,000	18, 34, 50	3.5 × 3.5 × 3.5	76 × 76 × 32	83	NA	37.34 ± 11.04	136.16 ± 57.94
infant (i)	11	2,000	13, 26, 39	3.5 × 3.5 × 3.5	64 × 64 × 39	83	NA	14.65 ± 2.39	27.99 ± 4.45
infant (ii)	7	1,500	15, 31, 46	2.9 × 2.9 × 2.9	64 × 64 × 48	83	2	18.05 ± 2.76	34.51 ± 7.67
infant (iii)	40	1,000	15, 37, 59	2.5 × 2.5 × 2.5	80 × 80 × 44	52	4	20.83 ± 1.87	34.69 ± 6.12

* DVARS, the root mean square of the temporal change of the fMRI voxel-wise signal at each time point.

### Functional connectivity matrix construction

Preprocessed fetal and infant data sets, combined in a singular template space, were masked by a gray matter mask and then submitted to the SLIC toolbox [61,62] (https://www.nitrc.org/projects/slic/) to generate a data-driven, group-balanced functional atlas consisting of 195 functional parcels and 8 networks. For each scan session, the average time series across voxels within each parcel were calculated and used to construct 195 × 195 RSFC matrices based on Pearson’s correlation coefficients.

For further graph analysis, we computed a graph adjacency matrix by thresholding the ROI-to-ROI correlation matrix using a relative threshold of the top 15%. Based on this adjacency matrix, the shortest path distance between 2 ROIs is defined as the minimum number of edges traversed in the optimal path between them. We then estimated the graph theory measures of GE and LE for each subject in CONN. For each ROI, we defined global efficiency as the average inverse shortest path distance from node n to all other nodes in the graph, and local efficiency as the average efficiency across all nodes in the local subgraph of node n (the subgraph consisting only of neighboring nodes).

### Harmonization on MRI condition effects

To account for non-biological variance introduced by different MRI conditions (i.e., in utero versus ex utero effects) and acquisition protocols (such as different time and spatial resolution), we applied a longitudinal harmonization technique to our RSFC measures using the R package LongComBat [36]. This tool has demonstrated superior efficacy in detecting longitudinal changes compared to an alternative harmonization method, cross-sectional ComBat, while also providing better control of type I error rates than unharmonized data that incorporates scanner as a covariate [36]. Since the harmonization is performed on model residuals, the longitudinal ComBat model matches the model in the final regression analysis. S6 Fig shows the model residuals before and after the harmonization protocol and the trajectory plot of RSFC before and after the harmonization.

### Statistical analysis

Linear regression models were used to test age effects for both RSFC and for graph measures (GE and LE), controlling for repeat measures. Regression analyses were thresholded at *p* < 0.05, FDR corrected. Average RSFC, GE, and LE that exhibited significant positive or negative associations with age (RSFC connection threshold *p* < 0.05) were then extracted to derive growth plots. Growth trajectory was modeled with the GAMMs in R using the “*gamm4*” function on average positively or negatively age-related RSFC, GE, LE, and for each within and between network RSFC pair. The GAMM model followed the equation RSFC (or graph measures) ~ 1 + s(Age) + (1|ID). We then calculated the derivative of each growth curve at 1/5th-week intervals to show the rate of change in RSFC over time.

In follow-up analyses, we repeated age-regression procedures described above, within fetal and infant data sets, separately. These secondary analyses served to isolate significant RSFC developmental patterns that may be present within each period, but masked when evaluating longer durations, here, also, spanning birth.

## Ethics statement

The Wayne State University Institutional Review Board approved all study procedures of the Perinatal Imaging of Neural Connectivity (PINC) project (IRB number: IRB-21-08-3875). Informed written consent was provided by participating pregnant people. This study was conducted according to the principles expressed in the Declaration of Helsinki. All analyses conducted in this work were approved by NYU Langone Health IRB (IRB number: i18-00960_MOD04).

## Supporting information

S1 Fig The gray matter mask and the functional atlas used in our RSFC analysis. Preprocessed fetal and infant data sets were masked by a gray matter mask and then submitted to the SLIC toolbox (https://www.nitrc.org/projects/slic/) to generate a data-driven, group-balanced functional atlas consisting of 195 functional parcels, as shown in S1 Fig. The gray matter mask was adapted from dHCP infant tissue template.
(PNG)

S2 Fig The distribution of age-related resting-state functional connectivity (RSFC) based on Euclidean distance. The Euclidean distance of RSFC is computed using the square root of the sum of squared differences between the central coordinates of corresponding ROIs. In this figure, regions with positive age-related RSFC are depicted in red, while those with negative age-related RSFC are depicted in blue. The figure does not exhibit a discernible contrast between the positively and negatively age-related RSFC, suggesting that distance may not be a significant factor influencing the increase or decrease of RSFC with age.
(PNG)

S3 Fig TSNR analysis. We conducted tSNR analyses on fetal and infant data sets, and the resulting plot of average SNR across networks is provided here. When we analyzed the signal and noise separately, we found that the mean signal levels (middle column in the figure below) are quite comparable between the fetal and infant data sets. However, the fetal data exhibits higher variance, indicating increased noise. This outcome aligns with the challenges typical in fetal imaging.
(PNG)

S4 Fig Quality control analysis of the final sample. This figure shows that the average RSFC-motion-distance correlation is negligible (< ± 0.2).
(PNG)

S5 Fig A lollipop plot and a distribution plot of scan ages. The lollipop plot displays each participant as a row, with points indicating visit times, color-coded as shown in the legend. Males are represented in blue, and females in pink. Separate density plots for participant ages are also provided for both fetal and infant MRI scans.
(PNG)

S6 Fig Effects of LongCombat Harmonization. (A) Additive effects of different sequences before and after applying LongCombat. (B) Comparison of positively age-related RSFC before and after LongCombat. (C) Comparison of negatively age-related RSFC before and after LongCombat.
(PNG)

S1 Table GAMM regression table for term s(Age). (DOCX)

S1 Data Data used to generate Figs 1D, 3B, 3D, and 4. Global-level resting-state functional connectivity (RSFC) values from 184 observations, as well as age and scan information are provided. Column A: Subject ID. Column B: Session ID. Column C: Scan condition (fetal vs. infant). Column D: gestational age at scan. Column E: Scan sequence ID. Column F: Averaged RSFC across all connectivity edges that show increasing RSFC with age. Column G: Averaged RSFC across all connectivity edges that show decrease RSFC with age. Fig 1D plots the data in Column F and G. Column H: Averaged global efficiency across all nodes that show increasing global efficiency with age. Column I: Averaged global efficiency across all nodes that show negative global efficiency with age. Fig 3B plots the data in Column H and I. Column J: Averaged local efficiency across all nodes that show increasing local efficiency with age. Fig 3D plots the data in Column J. Column K and L involved age-related RSFC results evaluated within the fetal period. Fig 4A plots the data in Column K and L. Column M and N involved age-related RSFC results evaluated within the infant period. Fig 4B plots the data in Column M and N.
(CSV)

S2 Data Data used to generate Fig 2. Network-level RSFC values before Combat harmonization, as well as age are provided. Column A: observation ID. Column B: Subject ID. Column C: gestational age at scan. Column D: Scan sequence ID. Column E to AN: 36 pairs of RSFC within and between 8 network, following the order: “RS-RS,” “RS-LS,” “RS-LT,” “RS-Occ,” “RS-Sub,” “RS-Inf,” “RS-Sup,” “RS-RT,” “LS-LS,” “LS-LT,” “LS-Occ,” “LS-Sub,” “LS-Inf,” “LS-Sup,” “LS-RT,” “LT-LT,” “LT-Occ,” “LT-Sub,” “LT-Inf,” “LT-Sup,” “LT-RT,” “Occ-Occ,” “Occ-Sub,” “Occ-Inf,” “Occ-Sup,” “Occ-RT,” “Sub-Sub,” “Sub-Inf,” “Sub-Sup,” “Sub-RT,” “Inf-Inf,” “Inf-Sup,” “Inf-RT,” “Sup-Sup,” “Sup-RT,” “RT-RT.” RS, R sensorimotor; LS, L sensorimotor; LT, L temporal; Occ, Occipital and Cerebellum; Sub, Subcortical; Inf, Inferior frontal; Sup, Superior frontal; RT, R temporal.
(CSV)

## Abbreviations:

BET: Brain Extraction Tool
BOLD: blood oxygen level-dependent
CNN: convolutional neural network
FD: framewise displacement
fMRI: functional magnetic resonance imaging
GA: gestational age
GE: global efficiency
GAMM: generalized addictive mixed-effect model
ICA: Independent Component Analysis
LE: local efficiency
PINC: Perinatal Imaging of Neural Connectivity
ROI: region of interest
RSFC: resting-state functional connectivity
tSNR: temporal signal-to-noise ratio
WSU: Wayne State University
