# Supplementary figures and images for: Trajectories of human brain functional connectome maturation across the birth transition
Source: PLoS Biol. 2024 Nov 19;22(11):e3002909. doi: 10.1371/journal.pbio.3002909 (PMC11575827; doi:10.1371/journal.pbio.3002909)

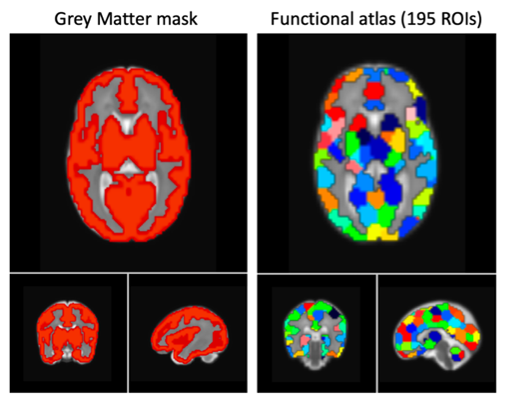

Supplement: S1 Fig — Preprocessed fetal and infant data sets were masked by a gray matter mask and then submitted to the SLIC toolbox (https://www.nitrc.org/projects/slic/) to generate a data-driven, group-balanced functional atlas consisting of 195 functional parcels, as shown in S1 Fig. The gray matter mask was adapted from dHCP infant tissue template. (PNG) [file pbio.3002909.s001.png]

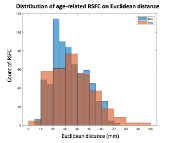

Supplement: S2 Fig — The Euclidean distance of RSFC is computed using the square root of the sum of squared differences between the central coordinates of corresponding ROIs. In this figure, regions with positive age-related RSFC are depicted in red, while those with negative age-related RSFC are depicted in blue. The figure does not exhibit a discernible contrast between the positively and negatively age-related RSFC, suggesting that distance may not be a significant factor influencing the increase or decrease of RSFC with age. (PNG) [file pbio.3002909.s002.png]

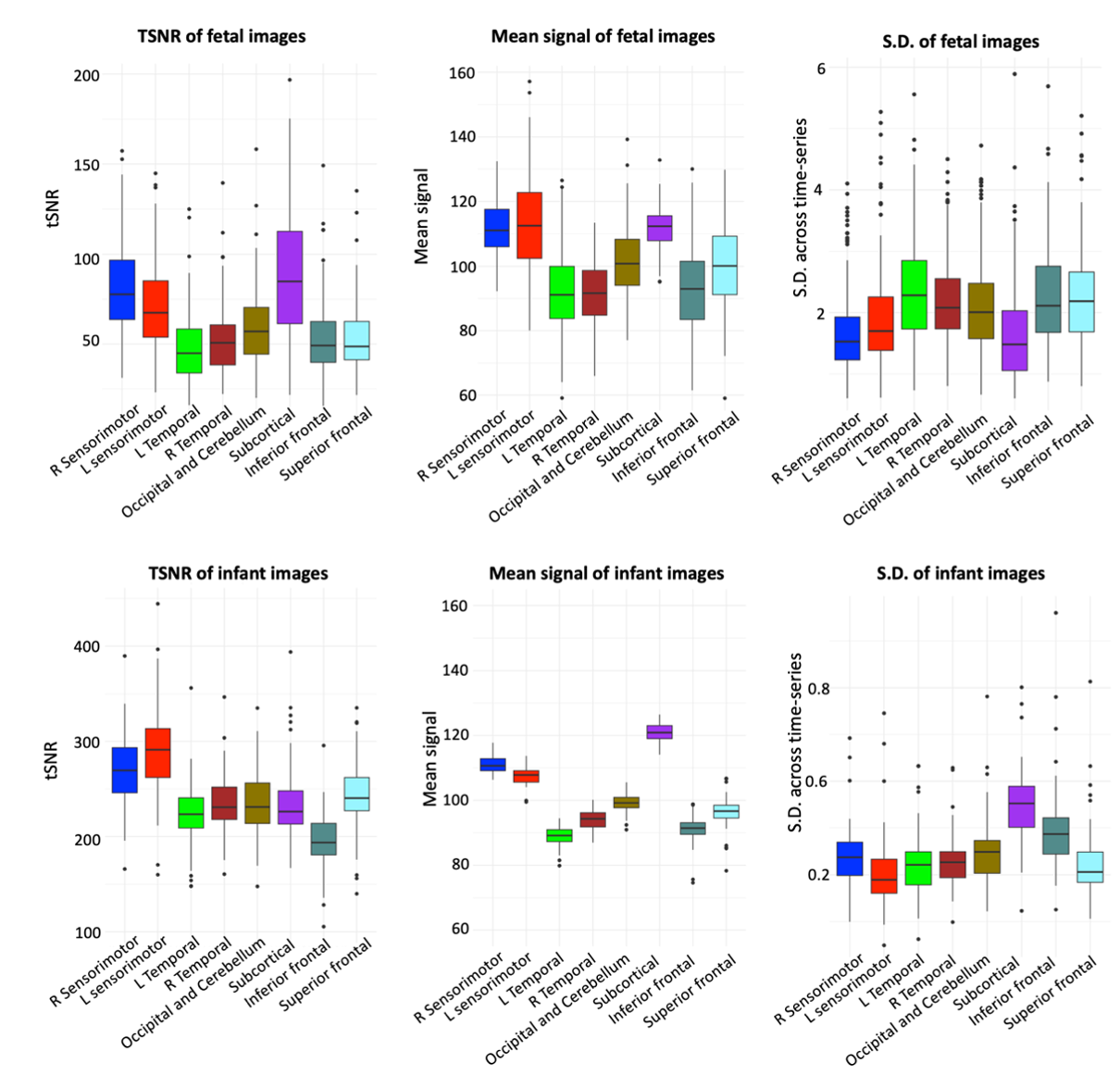

Supplement: S3 Fig — We conducted tSNR analyses on fetal and infant data sets, and the resulting plot of average SNR across networks is provided here. When we analyzed the signal and noise separately, we found that the mean signal levels (middle column in the figure below) are quite comparable between the fetal and infant data sets. However, the fetal data exhibits higher variance, indicating increased noise. This outcome aligns with the challenges typical in fetal imaging. (PNG) [file pbio.3002909.s003.png]

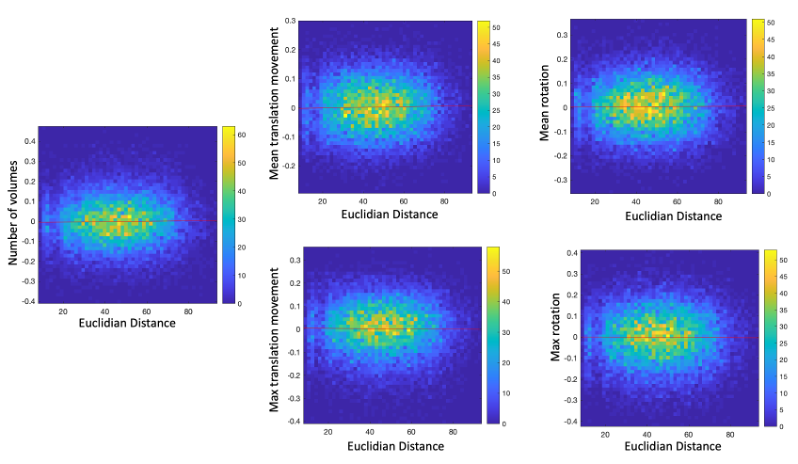

Supplement: S4 Fig — This figure shows that the average RSFC-motion-distance correlation is negligible (< ± 0.2). (PNG) [file pbio.3002909.s004.png]

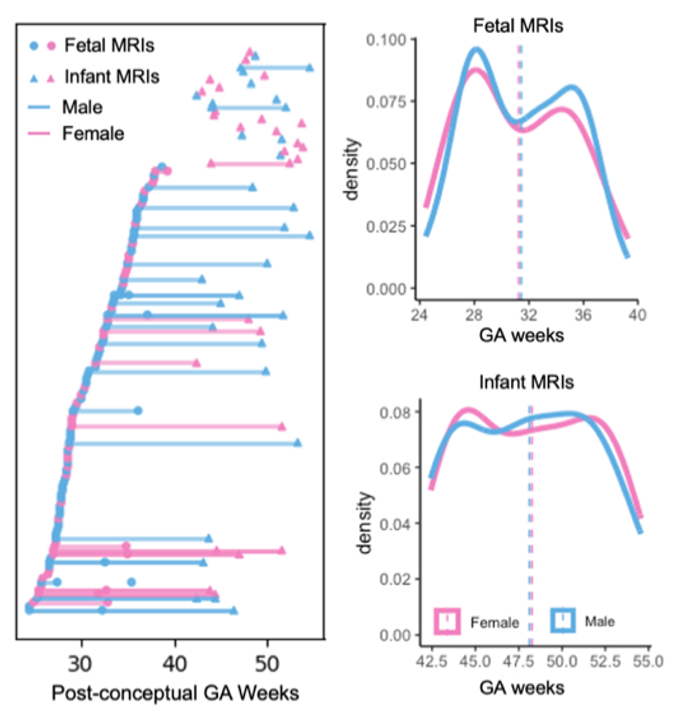

Supplement: S5 Fig — The lollipop plot displays each participant as a row, with points indicating visit times, color-coded as shown in the legend. Males are represented in blue, and females in pink. Separate density plots for participant ages are also provided for both fetal and infant MRI scans. (PNG) [file pbio.3002909.s005.png]

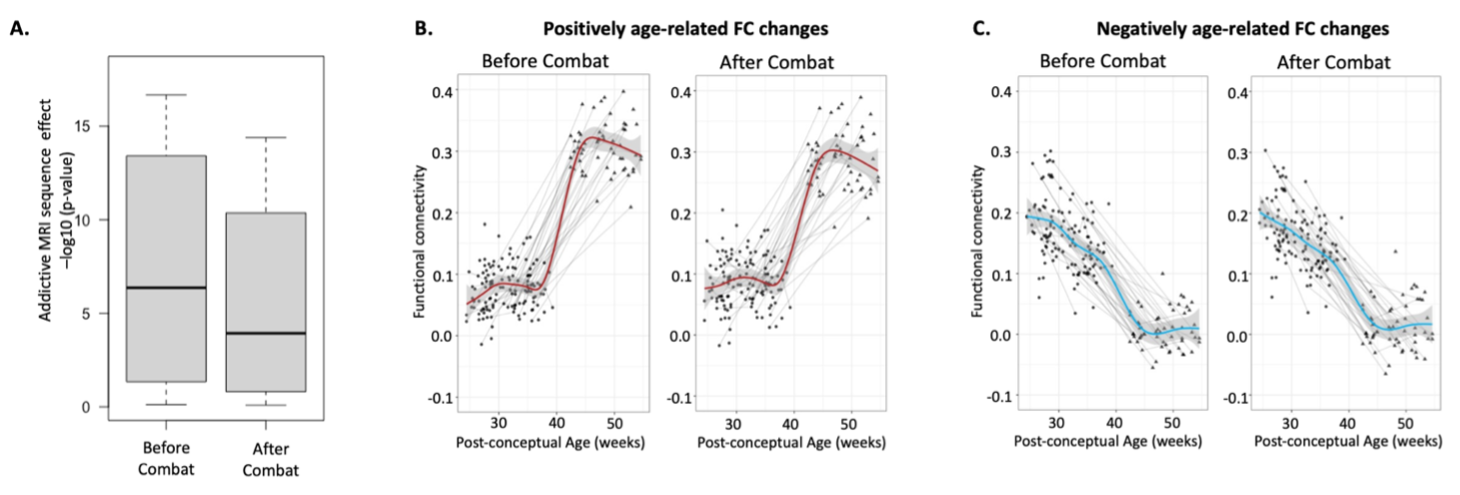

Supplement: S6 Fig — (A) Additive effects of different sequences before and after applying LongCombat. (B) Comparison of positively age-related RSFC before and after LongCombat. (C) Comparison of negatively age-related RSFC before and after LongCombat. (PNG) [file pbio.3002909.s006.png]
