# Supplementary material for: Trajectories of human brain functional connectome maturation across the birth transition
Source: PLoS Biol. 2024 Nov 19;22(11):e3002909. doi: 10.1371/journal.pbio.3002909 (PMC11575827; doi:10.1371/journal.pbio.3002909)
Supplement: S1 Table — (DOCX) [file pbio.3002909.s007.docx]

| **Supplementary Table S1. GAMM regression table for term s(Age)** | | | | | | | |
| --- | --- | --- | --- | --- | --- | --- | --- |
|  |  | | Statistics (*F*) | | p | |  |
| Global measures | | |  | |  | |  |
|  | Average positively age-related RSFC | 119.5 | | | <2e-16 *** | | |
|  | Average negatively age-related RSFC | 88.89 | | | <2e-16 *** | | |
|  | Average positively age-related GE | 67.48 | | | <2e-16 *** | | |
|  | Average negatively age-related GE | 44.18 | | | <2e-16 *** | | |
|  | Average positively age-related LE | 12.86 | | | <2e-16 *** | | |
| Network measures | |  | | |  | | |
|  | R Sensorimotor - R Sensorimotor | 12.96 | | | <2e-16 *** | | |
|  | R Sensorimotor - L Sensorimotor | 35.13 | | | <2e-16 *** | | |
|  | R Sensorimotor - L Temporal | 9.877 | | | 3.13e-06*** | | |
|  | R Sensorimotor - Occipital and Cerebellum | 91.33 | | | <2e-16 *** | | |
|  | R Sensorimotor - Subcortical | 4.886 | | | 0.0381 * | | |
|  | R Sensorimotor - Inferior frontal | 31.19 | | | <2e-16 *** | | |
|  | R Sensorimotor - Superior frontal | 0.112 | | | 0.739 | | |
|  | R Sensorimotor - R Temporal | 2.849 | | | 0.182 | | |
|  | L Sensorimotor - L Sensorimotor | 8.286 | | | 0.004 ** | | |
|  | L Sensorimotor - L Temporal | 2.121 | | | 0.095 | | |
|  | L Sensorimotor - Occipital and Cerebellum | 0.237 | | | 0.627 | | |
|  | L Sensorimotor - Subcortical | 21.2 | | | 8.14e-06 *** | | |
|  | L Sensorimotor - Inferior frontal | 24.93 | | | 1.55e-06 *** | | |
|  | L Sensorimotor - Superior frontal | 29.39 | | | <2e-16 *** | | |
|  | L Sensorimotor - R Temporal | 26.19 | | | <2e-16 *** | | |
|  | L Temporal - L Temporal | 5.036 | | | 0.004 ** | | |
|  | L Temporal - Occipital and Cerebellum | 10.79 | | | 1.27e-04 *** | | |
|  | L Temporal - Subcortical | 29.67 | | | <2e-16 *** | | |
|  | L Temporal - Inferior frontal | 14.22 | | | 2.20e-04 *** | | |
|  | L Temporal - Superior frontal | 7.883 | | | 3.12e-04 *** | | |
|  | L Temporal - R Temporal | 52.52 | | | <2e-16 *** | | |
|  | Occipital and Cerebellum - Occipital and Cerebellum | 63.96 | | | <2e-16 *** | | |
|  | Occipital and Cerebellum - Subcortical | 38.86 | | | <2e-16 *** | | |
|  | Occipital and Cerebellum - Inferior frontal | 7.174 | | | 0.008 ** | | |
|  | Occipital and Cerebellum - Superior frontal | 0.412 | | | 0.522 | | |
|  | Occipital and Cerebellum - R Temporal | 41.65 | | | <2e-16 *** | | |
|  | Subcortical - Subcortical | 42.79 | | | <2e-16 *** | | |
|  | Subcortical - Inferior frontal | 3.566 | | | 0.011 * | | |
|  | Subcortical - Superior frontal | 8.55 | | | 0.001 ** | | |
|  | Subcortical - R Temporal | 12.94 | | | 4.15e-04 *** | | |
|  | Inferior frontal - Inferior frontal | 16.48 | | | 7.35e-05 *** | | |
|  | Inferior frontal - Superior frontal | 18.52 | | | <2e-16 *** | | |
|  | Inferior frontal - R Temporal | 24.72 | | | <2e-16 *** | | |
|  | Superior frontal - Superior frontal | 23.84 | | | <2e-16 *** | | |
|  | Superior frontal - R Temporal | 13.14 | | | <2e-16 *** | | |
|  | R Temporal - R Temporal | 20.03 | | | <2e-16 *** | | |
| Signif. codes: 0 *** 0.001 ** 0.01 * 0.05 | | | |  | |  | |
